# Impact of dietary zinc nanoparticles and probiotics on broiler health and productivity

**DOI:** 10.5455/javar.2025.l868

**Published:** 2025-03-23

**Authors:** Amera N. Alqahtani

**Affiliations:** Department of Biological Sciences, College of Science, University of Jeddah, Jeddah, Saudi Arabia

**Keywords:** ZnNPs, probiotics, growth, blood indicators, microbiota, broilers

## Abstract

**Objective:**

This study investigates the effects of dietary nano-zinc (ZnNPs), probiotics (P), and their combined use such as growth promoters, antibacterial agents, and organic antioxidants on the growth performance, carcass characteristics, blood biochemistry, meat quality, and cecal microbiota of broiler chicks.

**Materials and Methods:**

The trial was conducted from 7 to 35 days of age using a randomized complete trial design with 240 unsexed Ross 308 chicks (initial body weight 104.37 ± 0.16 gm). The chicks were allocated into four treatment groups, each containing 60 chicks, with six replicates (4×6×10). The treatments were as follows: a control group fed a standard diet and three experimental groups receiving diets supplemented with ZnNPs (3.0 cm³/kg), probiotics (P, 2.0 cm³/kg), or a combination of ZnNPs + P (3.0 + 2.0 cm³/kg).

**Results:**

The study revealed that ZnNPs and probiotics exhibited significant antibacterial activity against harmful bacteria and mold, effectively suppressing microbial growth at concentrations ranging from 50 to 95 µg/ml. The inclusion of ZnNPs and probiotics in the diets resulted in improved growth performance, with a higher body weight compared to the control group (*p* < 0.05). All carcass characteristics were positively influenced by the treatments, showing significant improvements compared to the control group (*p *< 0.05). Furthermore, the levels of malondialdehyde (MDA) were significantly reduced in the ZnNPs and probiotics-treated groups, suggesting enhanced antioxidant activity (*p *< 0.05). Blood biochemical indicators, including immunoglobulin concentrations, were higher in the treated groups, pointing to an improved immune response. The meat quality of the broilers also significantly improved in terms of texture, juiciness, and tenderness. Additionally, the number of harmful cecal microorganisms decreased in the supplemented groups, leading to a healthier gut microbiota and overall improved animal health.

**Conclusion:**

The study shows that dietary supplementation with ZnNPs and probiotics has a positive impact on broiler growth, carcass traits, meat quality, blood parameters, and microbial status. These results suggest that incorporating ZnNPs and probiotics into broiler diets can be an effective strategy for enhancing productivity, meat quality, and overall health status, ultimately improving the sustainability of poultry production systems.

## Introduction

Recently, there has been a significant global increase in demand for meat, eggs, and dairy products from animals and birds that feed on organic materials [1–4]. In the poultry sector, nutritional supplements are essential, as feed costs constitute 60%–70% of total production expenses [5,6]. Livestock and poultry have widely employed natural feed dietary supplements, often incorporating artificial growth stimulants and antimicrobial substances, to enhance their growth productivity and carcass quality [7,8]. Researchers are progressively exploring alternatives to antibiotic growth promoters to improve chicken well-being, quality, and production effectiveness [9,10]. As a result, researchers are integrating herbs and phytogenic naturals as active dietary elements to enhance avian immunity as well as efficiency [11,12].

Various techniques for nanoparticle creation, including biosynthesis, are renewable, safe, and compatible with life. Chemical and physical processes often require hazardous solvents or energy sources [13]. NPs offer excellent qualities such as strong adsorption capacity, enhanced catalytic efficiency, and extensive surface area, with indications of increasing adsorption [14]. Zinc (Zn) significantly impacts bird functions such as carbohydrates, proteins, lipid metabolism, immune regulation, hormone production, nucleic acid and protein production, and antioxidants [15]. In broilers, the NRC [16] specifies a zinc need of 40 ppm; however, feed manufacturers typically incorporate an additional 100–120 ppm of zinc in commercial diets to maximize growth. Feng et al. [17] suggest that increasing Zn intake could potentially lead to decreased costs, increased metal secretion in manure, ecological defilement, and decreased vitamin solidity [18]. Due to their influence on bird metabolic processes and health outcomes, zinc oxide nanoparticles have gained recognition as a novel feed addition for enhancing health [19]. Earlier research showed that adding ZnNPs to feed could enhance growth efficiency, such as body weight gain (BWG), feed conversion rate (FCR), meat quality, and egg production. It could also help the immunity and the community of microbes in the gut [5].

Other than commercial antibiotics, non-specific immunomodulators such as polysaccharides, organic acids, enzymes, essential oils, probiotics, prebiotics, symbiotics, postbiotics, and synbiotics have become popular to improve the gut microbiota of chickens [7,20,21]. Probiotics in animal diets enhance growth, production performance, disease prevalence, immunity, digestibility, and fecal microflora in poultry [22]. Probiotics promoted beneficial growth and reproductive outcomes, influenced gut histomorphology and immunology, and increased beneficial microbiota [23]. Furthermore, Abou-Kassem et al. [24] found that dietary probiotics significantly influenced carcass aspects in quail meat, including color, redness, a*, L* values, and coliform, compared to the control. Probiotics activate local cell-mediated immunity in birds, reducing antimicrobial use and antibiotic residue in food animals and thus preventing the spread of antibiotic resistance [25]. However, while several studies have explored the beneficial effects of ZnNPs or probiotics individually, their synergistic effects remain unexplored. Thus, we hypothesized that the combination may provide more beneficial effects in promoting growth, preventing pathogenic bacteria, and boosting immunity and antioxidant status in broilers. With this sense, this research sought to investigate the use of zinc biological NPs and probiotics, individually or in combination, as growth enhancers, antibacterial agents, and antioxidant supplements in the nutrition of Ross 308 broilers, focusing on production rates, carcass quality, blood serum parameters, meat quality, and microbiota composition.

## Materials and Methods

### Ethical approval

The study conforms to the guidelines for utilizing experimental animals set forth by the Department of Biological Sciences, College of Science, University of Jeddah, Jeddah 21589, Saudi Arabia.

### Bacterial isolates and biosynthesis of ZnNPs

*Bacillus subtilis* AM12, utilized for the biosynthesis of ZnNPs, was extracted from soil specimens, as reported by Kannan et al. [26]. ZnNPs were synthesized by combining 10 ml of *B*. *subtilis* AM12 supernatant with 90 ml of zinc nitrate (1 mmol) under optimum circumstances, followed by incubation in a moving incubator at 130 rpm and 30°C for 72 h. The clear change from a colorless liquid to white shows that zinc nitrate is being turned into ZnNPs by the *B*.* subtilis* AM12 biomass [27]. The ZnNPs were provided by Nano Gate Company (Cairo, Egypt). They have a spherical shape, are less than 30 nm in size, and have a purity of approximately 99.9%. These nanoparticles are used for biomedical and other applications.

### Microbial strains

The microbial strains used in this study were provided by the Biology Department, College of Science, University of Jeddah. All bacterial and fungal strains were identified by the staff members of the Biology Department. This study utilized bacterial strains (*Bacillus cereus*, *Listeria monocytogenes*, *Streptococcus pyogenes*, *Escherichia coli*, *Salmonella typhi*, and *Pseudomonas aeruginosa*) and fungal strains (*Alternaria alternata*, *Aspergillus flavus*, *Fusarium oxysporum*, *Aspergillus niger*, *Penicillium solitum*, and *Penicillium crustosum*) to detect the bacterial efficacy of ZnNPs.

### Disc assay

The antimicrobial activity of ZnNPs was assessed utilizing the disc diffusion method [28]. Mueller–Hinton agar (MHA) plates were inoculated with 0.1 of antimicrobial inoculum, whereas Sabouraud dextrose agar (SDA) plates were inoculated with fungal mycelium. The inoculated MHA and SDA plates were inoculated with paper discs saturated with varying levels of ZnNPs (100, 150, 200, 250, and 300 µg/ml). The MHA plates were incubated at 37°C for 24 h, whereas the SDA plates were incubated at 28°C for five days. The measured zones of inhibition (mm) were recorded [29].

### Minimum inhibitory concentration (MIC), minimum bactericidal concentration (MBC), and minimum fungicidal concentration (MFC) estimation

The MIC was ascertained utilizing the micro-dilution broth technique as outlined by “the European Committee on Antimicrobial Susceptibility Testing” [30]. Tubes holding 9 ml of MHB or SDB were vaccinated with 0.1 ml of bacterial inoculum or a basic fungal spore suspension (3×10³ CFU/ml), followed by the addition of 50 µl of ZnNPs at doses of 100, 150, 200, 250, and 300 µg/ml. Free ZnNP tubes were monitored. All MH tubes were incubated at 37ºC for 24 h, whereas SD tubes were incubated at 28ºC for five days. The MIC exhibited a minimal dose of ZnNPs, which inhibited microbial growth. Conversely, MBC and MFC were assessed according to Aldalin et al. [31] by distributing a series of MIC tubes onto fresh MHA or SDA plates, followed by incubation under established conditions and subsequent observation of bacterial or mold growth. The minimum level that completely eradicates bacterial or mold growth is referred to as MBC or MFC [32].

### Birds, design, and diets

One-week-old Ross 308 broiler chicks, each weighing 104.50 ± 0.10 gm, were used in an experiment conducted with a completely randomized design. The chicks were divided into 4 groups of 60 birds each, with six replicates (4×6×10). The birds were reared on the floor, with each replicate placed in an area measuring (50×100×100 cm). Temperature, humidity, and lighting were controlled automatically, with a lighting schedule of 23 h of light and 1 h of darkness.

The concentrations of biological ZnNPs and P solutions were 500 mg/l of ZnNPs and 1.5 × 108 CFU/ml of P, respectively. The experimental groups received primary feed, while the other groups received rations containing ZnNPs, *p*, or a combination of ZnNPs and *p* at 3.0, 2.0, and 3.0 + 2.0 cm³/kg feed, respectively. The total chicks received the primary diets according to NRC [16] for 7–35 days, as illustrated in Table 1. The experimental diets were given in two phases: starter (2–3 weeks) and finisher (3–5 weeks). All chicks were kept under the same environmental, management, and health conditions.

### Growth performance and carcass traits

Chicks were weighed individually once a week. Average daily feed intake (FI), BWG, and FCR were calculated based on timing and accumulation data. Fodder shortages were documented throughout the day, and allocation was adjusted to accommodate feed demand.

Six chickens were chosen from each group in preparation for slaughter. The slaughter was assessed, and the consumable components “(liver, gizzards, and hearts), non-consumable components (spleen and bursa), and abdominal fat were noted as gm per kg of slaughter weight.” Dressed weight is calculated as (carcass weight + edible weight)/live weight.

### Blood biochemical

Blood specimens were obtained after slaughter from six birds per set, immediately prepared using a Janetzki T32c centrifuge at 5,000 rpm for 15 min, and subsequently frozen at –25°C until biochemical testing [33]. The serum levels of total protein (TP), albumin, globulin, alanine (ALT), aspartate aminotransferase (AST), triglyceride (TG), total cholesterol (TC), low-density lipoprotein (LDL), and very low-density lipoprotein (VLDL) were determined in accordance with the manufacturer’s instructions of the commercial kit (Spinreact Co., Spain) [34].

Furthermore, immunological responses, including immunoglobulin A (IgA), immunoglobulin Y (IgY), and immunoglobulin M (IgM), were assessed following the manufacturer’’s instructions for readily accessible kits. The serum concentrations of “glutathione (GSH), malondialdehyde (MDA), and the activities of glutathione reductase (GSR), glutathione S-transferase (GST), superoxide dismutase (SOD), and glutathione peroxidase (GPx)” were assessed following the manufacturer’s protocols from Cell Biolabs (San Diego, USA).

### Breast meat quality

The color indicators (L* for lightness, a* for redness, and b* for yellowness) of raw and cooked meat specimens (2 cm cubes) were evaluated using a Hunter Lab colorimeter (Flex EZ, USA) following the method described by Wattanachant et al. [35]. The shear force of 2 cm cooked pork cubes was measured using a texture analyzer (Compac-100 model, Sun Scientific Co., Tokyo, Japan) equipped with a crosshead and a load cell. The crosshead speed was set at 240 mm/min with a 10 kg load cell. The cutting edge applied vertical pressure to the muscle fibers of the tissue. The shear force ratio represented the peak value pattern of the shear force.

Lipid oxidation was assessed using the 2-thiobarbituric acid test (TBA) [36]. The total volatile base nitrogen (TVBN) was assessed following the methodology of Botta et al. [37]. The pH levels of minced beef samples were evaluated by utilizing a pH meter.

### Sensory assessing

The meat specimens were cubed, and eight panelists received them on foam plates labeled with random three-digit codes. The sensory panel evaluated hue, taste, look, and juiciness utilizing a 7-point hedonic scale, with tap water supplied between sessions to modify mouthfeel [38].

**Table 1.** Composition and chemical analysis of the starter and finisher basal diets as fed.

Items	Starter (2–3 weeks)	Finisher (4–5 weeks)
Ingredients %		
Yellow corn	55.89	57
Soybean meal 44%	31.5	29.5
Gluten meal 60%	6.5	4.83
Di calcium phosphate	1.7	1.7
Limestone	1.24	1.15
Vit-min premix^*^	0.3	0.3
NaCl	0.3	0.3
DL-methionine	0.13	0.0
L-lysine HCl	0.24	0.18
Choline 50%	0.2	0.2
Soybean oil	2.0	4.84
Total	100	100
Calculated analysis^**^:		
DM %	91.72	90.43
CP %	23.00	20.94
ME kcal/kg diet	2996.30	3150.70
Calcium %	1.00	0.96
Phosphorous (available) %	0.44	0.44
Lysine %	1.3	1.17
Methionine + cysteine %	0.90	0.70
CF %	3.52	3.38

CF, crude fiber; CP, crude protein; DM, dry matter; ME, metabolizable energy.

* Vitamin-mineral premix provided per kg diet: Vit. A, 12,000 IU; Vit. D3, 5,000 IU; Vit. E, 16.7 gm; Vit. K, 0.67 gm; Vit. B1, 0.67 gm; Vit. B2, 2 gm; Vit. B 6, 0.67 gm; Vit. B12, 0.004 gm; nicotinic acid, 16.7 gm; pantothenic acid, 6.67 gm; biotin, 0.07 gm; folic acid, 1.67 gm; choline chloride, 400 gm; Zn, 23.3 gm; Mn, 10 gm; Fe, 25 gm; Cu, 1.67 gm; I, 0.25 gm; Se, 0.033 gm and Mg, 133.4 gm.

** Calculated, according to NRC [16].

### Microbial count in the diet and cecal specimens

The dietary specimens underwent microbiological examination at intervals of 0, 7, 14, and 21 days. Diet specimens were combined with sterile saline peptone solution at a ratio of 1:10 (w/v) in a screw-cap jar and blended for 3 min. Various media were employed to quantify the microorganisms’ numbers. The total bacterial count (TBC) was determined using plate count agar incubated at 30°C for 48 h. The total yeast and mold count (TYMC) was assessed on Rose Bengal Chloramphenicol agar over five days at 25°C. *Coliforms* were enumerated on MacConkey agar medium subsequently. *Escherichia coli* was enumerated on Tryptone-Bile Glucuronide Agar at 37°C for 24 h.

The microbial counts in the broiler cecum were assessed in accordance with the diet. Cecal samples (five replicates) were homogenized in a screw-cap bottle with sterilized saline peptone solution (1:10, w/v). Decimal serial dilutions up to 107 were prepared. The various microorganisms were enumerated on designated media. The TBC was quantified on Plate Count Agar. Violet Red Bile Agar (Biolife, Italy) was employed for coliform enumeration following incubation at 37°C for 24 h. *Escherichia coli* was enumerated on Tryptone-Bile Glucuronide Agar at 37°C for 24 h. *S*.* spp*. was quantified on S.S. agar; the presence of black colonies indicates the detection of *S*.* spp.* Yeast and mold were quantified. MRS-medium was employed to enumerate lactic acid bacteria (LAB) as per Argyri et al. [39]. *E*.* spp*. was enumerated on *C*.* enterococci* agar; the presence of red colonies indicates its detection.

### Statistical analysis

To ensure data normality and homogeneity of variance, Shapiro–Wilk and Levene’s tests were conducted. The data (mean cage) were assessed utilizing one-way ANOVA with the GLM technique in SPSS. These types employed were as follows:

Y_ijk_ = μ + T_i_ + e_ij_,

where Y_ijk_ = observation; μ = overall mean; T_i_ = effect of Z, *p* individually, and their combined inclusion levels (3, 2); and e_ij_ = randomized error. The difference between the means was assessed using the Duncan test. The results are visible in the form of mean values and SEM. The statistical significance was determined at *p *< 0.05. The orthogonal polynomial contrasts were used to determine the linear (L) and quadratic (Q) effects.

## Results

### Antibacterial effect of ZnNPs

Table 2 illustrates the antimicrobial efficacy of ZnNPs toward 6 bacterial strains. The widths of the inhibitory zones of ZnNPs were raised in a concentration-related manner. The sizes of the inhibition zones for ZnNPs ranged from 12.7 to 24.5 mm. ZnNPs recorded the most extensive inhibitory zone diameters against *S*.* pyogenes*, measuring 24.5 mm. Consequently, *S*.* pyogenes* exhibited the highest sensitivity among the Gram-positive bacteria to the evaluated nanoparticles. However, *P*.* aeruginosa* had the highest resistance among Gram-negative bacteria to ZnNPs concentrations. The ZnNPs exhibited inhibition zone diameters against the investigated fungus ranging from 16.4 to 29.1 mm. *Aspergillus niger* had the highest sensitivity to NPs, and *P*.* crustosum* showed the greatest resistance. The ZnNPs suppressed microbial growth with MIC values from 50 to 95 µg/ml. No microbial proliferation was observed at doses ranging from 90 to 170 µg/ml for ZnNPs.

### Growth performance

Table 3 presents the impacts of ZnNPs and *p* on LBW, BWG, FI, FCR, and PI in chicks. ANOVA showed that treatments had a significant (*p* < 0.05) impact on LBW across all experimental groups at 2 and 5 weeks. Effects on BWG were seen at 1–2, 2–3, 4–5, and 1–5 weeks, and effects on FI and FCR were seen throughout the experiment (1–5 weeks) in contrast to the control. The ZnNPs + P and ZnNPs groups maintained optimal LBW throughout the whole test, while all groups containing ZnNPs and ZnNPs + P recorded superior BWG. The FI was markedly diminished in the ZnNPs + P and P groups relative to the control (*p *< 0.05). On the other hand, during the test period of 1–5 weeks, the FCR showed substantial differences among all treatments and the control (*p *< 0.05), with ZnNPs + P showing a better value than P and ZnNPs. Furthermore, the PI value was superior in the treatments relative to the control.

### Carcass criteria

Table 4 indicates that most carcass features were substantially affected (*p* < 0.05) by the feed treatment, excluding the proportion of the bursa. We observed optimal carcass, dressing, and giblet values for ZnNPs + P, P, and ZnNPs, respectively, compared to the control group. All nutritional groups led to a reduction in the percentage of abdominal fat compared to the control group.

**Table 2.** Antimicrobial activity of ZnNPs against tested bacteria and fungi represented by inhibition zones diameters (mm), MIC, MFC, and MBC.

Microorganisms	ZnNPs (*µ*g/ml)					ZnNPs	
Bacteria	100	150	200	250	300	MIC	MBC
*Bacillus cereus *	17.2 ± 0.6^b^	18.2 ± 0.2^b^	20.9 ± 0.1^b^	21.6 ± 0.5^b^	22.8 ± 0.4^b^	60^d^	110^e^
*Listeria monocytogenes *	16.5 ± 0.5^c^	17.5 ± 0.3^c^	19.5 ± 0.2^c^	20.5 ± 0.4^c^	23.4 ± 0.2^c^	75^cd^	130^d^
*Streptococcus pyogenes *	18.3 ± 0.6^a^	19.4 ± 0.5^a^	21.8 ± 0.3^a^	22.9 ± 0.2^a^	24.5 ± 0.3^a^	50^e^	90^f^
*Escherichia coli *	15.2 ± 0.8^d^	16.9 ± 0.6^d^	18.8 ± 0.1^d^	19.5 ± 0.7^d^	20.6 ± 0.7^d^	80^c^	140^c^
*Salmonella * *typhi*	14.3 ± 0.8^e^	15.2 ± 0.8^e^	17.2 ± 0.4^e^	18.4 ± 0.8^e^	19.8 ± 0.8^e^	85^b^	150^b^
*Pseudomonas aeruginosa *	12.7 ± 0.9^f^	14.8 ± 0.2^f^	16.4 ± 0.6^f^	17.5 ± 0.5^f^	18.5 ± 0.2^f^	95^a^	170^a^
	Fungi					MIC	MFC
*Alternaria alternata*	20.9 ± 0.1^c^	21.5 ± 0.1^c^	22.9 ± 0.2^c^	24.2 ± 0.1^c^	25.2 ± 0.5^c^	70^c^	120^d^
*Aspergillus flavus*	21.8 ± 0.1^b^	22.3 ± 0.2^b^	24.2 ± 0.3^b^	26.5 ± 0.2^b^	28.2 ± 0.2^b^	65^cd^	110^e^
*Fusarium oxysporum*	19.6 ± 0.2^d^	20.5 ± 0.5^d^	21.9 ± 0.4^d^	23.2 ± 0.4^d^	24.2 ± 0.3^d^	75^bc^	140^c^
*Aspergillus niger*	22.4 ± 0.3^a^	24.8 ± 0.4^a^	25.7 ± 0.9^a^	27.6 ± 0.5^a^	29.1 ± 0.4^a^	55^d^	100^f^
*Penicillium solitum*	18.5 ± 0.2^e^	19.5 ± 0.7^e^	20.8 ± 0.8^e^	22.6 ± 0.7^e^	23.4 ± 0.2^e^	80^b^	150^b^
*Penicillium crustosum*	16.4 ± 0.3^f^	18.2 ± 0.2^f^	19.5 ± 0.4^f^	21.4 ± 0.8^f^	22.6 ± 0.1^f^	95^a^	170^a^

The mean in the same column with different lowercase letters is significantly different between bacterial and fungal strains’ tolerance against ZnNPs (*p ≤ 0.05*).

MBC = minimum bactericidal concentration; MFC = minimum fungicidal concentration; MIC = minimum inhibitory concentration.

**Table 3.** Effect of dietary ZnNPs and probiotics on broiler growth performance.

Traits	Age (weeks)	Treatments				SIM	*p* value^1^		
		Control	ZnNPs	P	ZnNPs + P		T	L	Q
BW (gm)	1	104.20	104.40	104.90	104.00	0.16	0.243	2.55	1.65
	2	302.69^c^	314.72^b^	337.09^a^	326.00^a^	4.12	0.001	0.007	0.021
	3	667.41	642.36	652.45	668.18	5.37	0.276	0.165	0.529
	4	1113.81	1103.36	1100.68	1132.72	8.03	0.545	0.450	0.726
	5	1843.30^b^	2161.86^a^	2084.09^a^	2143.42^a^	40.07	0.000	0.001	0.002
BWG (gm/bird/day)	1–2	28.36^c^	30.04^b^	33.17^a^	31.71^a^	0.57	0.000	0.006	0.018
	2–3	40.52^a^	36.40b^c^	35.04^c^	38.02^ab^	0.70	0.007	0.007	0.675
	3–4	74.40	76.83	74.70	77.42	3.32	0.311	0.309	0.138
	4–5	81.5^c^	117.61^a^	109.27^b^	112.30^ab^	3.38	0.000	0.000	0.001
	1–5	56.08^b^	65.22^a^	63.04^a^	64.86^a^	1.16	0.000	0.001	0.004
FI (gm/bird/day)	1–2	39.30^a^	37.34^b^	38.27^ab^	38.81^ab^	0.39	0.110	0.244	0.110
	2–3	70.74^a^	55.42^b^	54.13^b^	56.87^b^	2.19	0.001	0.006	0.308
	3–4	124.23^a^	118.04^b^	112.03^c^	117.72^b^	1.41	0.001	0.004	0.084
	4–5	116.48^b^	148.56^a^	131.65^ab^	136.11^a^	4.16	0.020	0.015	0.020
	1–5	87.69^ab^	89.84^a^	84.02^b^	86.88^ab^	0.88	0.119	0.094	0.036
FCR (feed/gain)	1–2	1.39^a^	1.24^b^	1.15^c^	1.16^c^	0.02	0.000	0.006	0.710
	2–3	1.74^a^	1.52^b^	1.54^b^	1.49^b^	0.03	0.038	0.172	0.217
	3–4	1.67^a^	1.54^b^	1.50^b^	1.52^b^	0.02	0.017	0.094	0.849
	4–5	1.43^a^	1.26^b^	1.20^b^	1.21^b^	0.03	0.027	0.210	0.842
	1–5	1.56^a^	1.39^b^	1.35^b^	1.35^b^	0.02	0.002	0.041	0.475
PI	5	118.24^b^	155.54^a^	154.29^a^	159.16^a^	5.31	0.000	0.006	0.044

BW, body weight; BWG, body weight gain; FCR, feed conversion ratio; FI, feed intake; P = probiotics 2 cm^3^; ZnNPs = zinc nanoparticles 3 cm^3^; ZnNPs + P = ZnNPs 3 cm^3^ + P 2cm^3.^

^a,b,c^Means within a row followed by different superscripts are significantly different (*p *≤ 0.05).^1^T, overall effects of treatments; L, linear effects of increasing treatment levels of broiler; Q, quadratic effects of increasing treatment levels of broiler.

**Table 4.** Carcass traits as affected by ZnNPs, probiotics, and their combination in broiler rations.

Traits	Treatments				SEM	*p value^1^*		
	Control	ZnNPs	P	ZnNPs + P		T	L	Q
Pre-slaughter weight	2010.00^bc^	2047.50^b^	1995.00^bc^	2240.00^a^	21.45	0.001	0.008	0.075
Carcass (%)	74.00^d^	74.49^c^	75.09^b^	75.58^a^	0.71	0.010	0.018	0.023
Giblets (%)	3.28^d^	4.20^a^	3.76^b^	3.60^c^	0.11	0.001	0.007	0.084
Dressing (%)	77.29^c^	78.69^b^	78.85^b^	79.19^ab^	0.23	0.006	0.047	0. 135
Abdominal fat (%)	1.11^a^	0.81^c^	0.38^d^	0.94^b^	0.07	0.002	0.027	0.287
Spleen (%)	0.12^b^	0.08^c^	0.12^b^	0.16^a^	0.01	0.017	0.085	0.346
Bursa (%)	0.10	0.12	0.14	0.13	0.01	0.135	0.207	0.087

P = probiotics 2 cm^3^; ZnNPs = zinc nanoparticles 3cm^3^; ZnNPs + P, ZnNPs 3cm^3^ + P 2cm^3^;

^a,b,c^Means within a row followed by different superscripts are significantly different (*p *≤ 0.05).

^1^T, overall effects of treatments; L, linear effects of increasing treatment levels of broiler; Q, quadratic effects of increasing treatment levels of broiler.

### Biochemical parameters

The nutritional treatment with ZnNPs + P, ZnNPs, and P significantly increased the concentrations of TP, albumin, globulin, calcium, IgA, IgM, and IgY, as well as the antioxidant enzymes GSH, GSR, GST, and SOD. Additionally, levels of amylase, protease, and lipase were also enhanced compared to the control group. When dietary supplements of ZnNPs + P and P were administered, levels of TC, TG, LDL, VLDL, and MDA were much lower than in the control group, as shown in Table 5.

### Meat quality

Adding ZnNPs and P to the broiler diet greatly improved the meat’s moisture, protein content, and pH (P and ZnNPs + P) relative to the control group, as shown in Table 6. Furthermore, the ZnNPs + P group significantly reduced the fat content of meat, TVBN, and TBA in comparison to the other groups. Furthermore, the treated groups considerably boosted the yellowness (b*), juiciness, tenderness, and flavor of the meat compared to the control group.

**Table 5.** Effect of diet supplementation by ZnNPs, P, and their combination on serum parameters of broiler.

Traits	Treatments				SEM	*p value^1^*		
	Control	ZnNPs	P	ZnNPs + P		T	L	Q
Biochemical								
Total protein (gm/dl)	4.50^c^	4.90^b^	6.50^a^	5.00^b^	0.27	0.001	0.007	0.021
Albumin (gm/dl)	3.20^c^	3.50^b^	4.00^a^	4.00^a^	0.14	0.004	0.413	0.351
Globulin (gm/dl)	2.10^c^	2.50^a^	2.40^b^	2.50^a^	0.16	0.006	0.127	0.094
Chloride (mmol/l)	105^a^	99^d^	100^c^	103^b^	1.64	0.010	0.017	0.847
Calcium (mmol/l)	2.20^d^	2.40^c^	3.20^a^	3.00^b^	0.17	0.001	0.007	0.021
Aspartate aminotransferase (U/I)	23^c^	25^bc^	35^a^	27^b^	1.89	0.000	0.023	0.648
Albumin, globulin, alanine (U/I)	20^c^	16^d^	25^a^	23^b^	1.28	0.001	0.249	0.362
Triglycerides (mmol/l)	1.20^a^	1.10^ab^	1.05^b^	1.00^b^	0.05	0.034	0.361	0.128
Cholesterol (mmol/l)	4.10^ab^	4.20^a^	4.20^a^	4.00^c^	0.03	0.023	0.015	0.372
Low-density lipoprotein (mmol/l)	3.40^ab^	3.50^a^	3.40^ab^	1.70^c^	0.34	0.000	0.142	0.460
Very low-density lipoprotein (mmol/l)	0.44^ab^	0.45^a^	0.28^c^	0.17^d^	0.03	0.000	0.026	0.710
Duodenal enzyme activity								
Amylase (U/l)	2830^d^	2935^c^	3145^b^	3750^a^	181.34	0.000	0.137	0.241
Protease (U/l)	130^fd^	140^c^	180^b^	190^a^	13.61	0.000	0.017	0.157
Lipase (U/l)	95^d^	100^c^	120^b^	130^a^	7.23	0.000	0.009	0.369
Oxidative enzymes								
Glutathione (mmol/l)	1.10^d^	1.30^b^	1.20^cb^	1.50^a^	0.93	0.001	0.006	0.308
Glutathione reductase (mmol/l)	1.30^d^	1.50^b^	1.35^cd^	1.80^a^	0.97	0.010	0.018	0.273
Glutathione S-transferase (mmol/l)	1.40^cd^	1.60^b^	1.31^d^	1.80^a^	0.13	0.002		
Superoxide dismutase (mmol/l)	1.50^d^	1.70^c^	1.90^b^	2.40^a^	0.17	0.000	0.006	0.365
Malondialdehyde (mmol/l)	17.90^a^	17.10^ab^	16.20^c^	16.20^c^	0.47	0.000	0.047	0.418
Immunoglobulin level								
Immunoglobulin A (ng/ml)	7.50^d^	7.80^c^	8.00^b^	8.50^a^	0.42	0.006	0.132	0.045
Immunoglobulin Y (ng/ml)	2.50^d^	2.90^c^	3.10^b^	3.50^a^	0.24	0.000	0.006	0.452
Immunoglobulin G (ng/ml)	13.00^d^	14.30^b^	16.00^a^	16.00^a^	0.67	0.000	0.024	0.310

P = probiotics 2 cm^3^; ZnNPs = zinc nanoparticles 3 cm^3^; ZnNPs + P = ZnNPs 3 cm^3^ + P 2 cm^3.^

^a,b,c^Means within a row followed by different superscripts are significantly different (*p* ≤ 0.05).

^1^T, overall effects of treatments; L, linear effects of increasing treatment levels of broiler; Q, quadratic effects of increasing treatment levels of broiler.

**Table 6.** Chemical, color parameters, and meat quality of broiler fed diet supplemented with ZnNPs, probiotics, and their combination in broiler rations.

Traits	Treatments				SEM	*p value^1^*		
	Control	ZnNPs	P	ZnNPs + P		T	L	Q
Chemical (%)								
Moisture	65.90^c^	64.3^d^	68.20^a^	67.10^b^	0.82	0.000	0.135	0.045
Protein	19.45^c^	20.12^b^	22.00^a^	21.20^b^	0.43	0.001	0.243	0.423
Lipids	14.10^ab^	15.00^a^	9.30^d^	11.50^c^	1.09	0.000	0.203	0.136
Ash	0.89^c^	1.10^a^	1.00^ab^	1.00^ab^	0.09	0.016	0.273	0.007
pH	5.50^c^	6.00^b^	6.00^b^	6.30^a^	0.14	0.002	0.013	0.317
TVBN	6.40^a^	5.90^b^	5.90^b^	5.50^c^	0.19	0.007	0.017	0.036
TBA	0.60^a^	0.60^a^	0.60^a^	0.30^c^	0.06	0.000	0.008	0.134
Color								
L^*^	60.10^a^	58.20^c^	60.0^ab^	59.60^b^	0.34	0.001	0.006	0.097
a^*^	6.00^c^	6.50^b^	6.00^c^	6.70^a^	0.12	0.012	0.046	0.243
b^*^	15.00^a^	15.00^a^	14.10^c^	14.80^b^	0.27	0.003	0.032	0.361
Sensorial								
Juiciness	4.20^bc^	4.05^c^	4.30^b^	4.40^a^	0.09	0.001	0.003	0.047
Tenderness	4.90^a^	4.75^b^	4.95^a^	4.70^c^	0.06	0.000	0.041	0.173
Taste	4.35^a^	4.20^b^	4.34^a^	4.20^b^	0.08	0.000	0.174	0.063
Aroma	4.50^ab^	4.50^ab^	4.55^a^	4.56^a^	0.04	0.001	0.037	0.196

a^*^ = redness; b^*^ = yellowness; L^*^ = lightness; P = probiotics 2 cm^3^; TBA = thiobarbituric acid; TVBN = total volatile basic nitrogen; ZnNPs = zinc nanoparticles 3 cm^3^; ZnNPs + P = ZnNPs 3 cm^3^ + P 2 cm^3^.

^a,b,c^Means within a row followed by different superscripts are significantly different (*p *≤ 0.05).

^1^T, overall effects of treatments; L, linear effects of increasing treatment levels of broiler; Q, quadratic effects of increasing treatment levels of broiler.

### Microbial load in dietary and cecal samples

Table 7 illustrates the bacterial count in feed and cecal specimens. The bacteria count was considerably lower (*p* < 0.05) in the treatments compared to the control. The interaction effect showed that ZnNPs + P performed better than the other treatments in reducing microbial counts in dietary samples, with a relative reduction of 12% in TBC, 25% in TYMC, 36% in *E*. *coli*, and 25% in coliform compared to the control group. The bacterial count increased over the feeding period.

**Figure 1.**
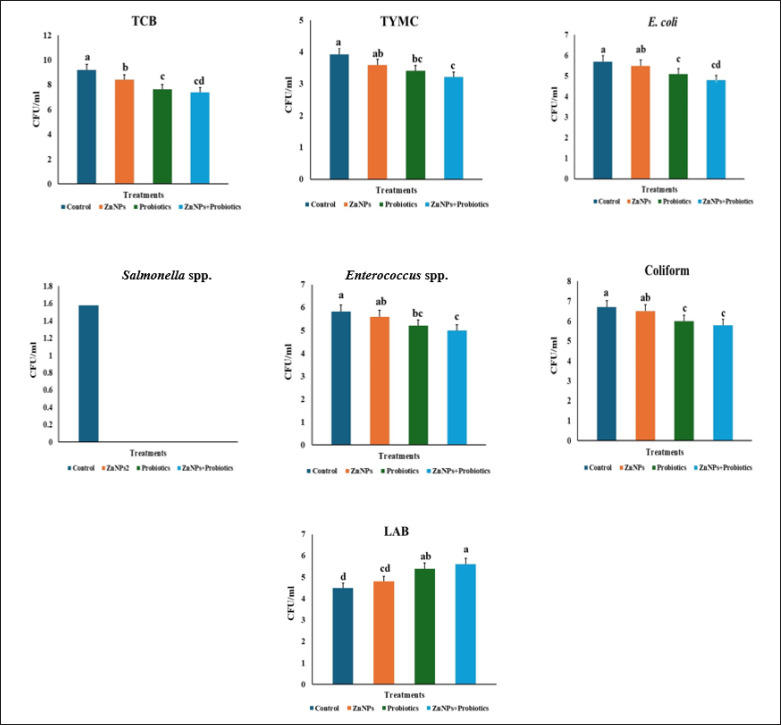
Effect of ZnNPs, probiotics, and their interaction on cecal microbiota (TBC, yeast and molds, *Escherichia coli*, coliform, *Salmonella*, *Enterococcus*, and LAB) represented by (Log CFU/ml) in broiler during feeding. *E*.* coli* = *Escherichia coli*; LAB = lactic acid bacteria; TBC = total bacterial count; TYMC = total yeast and mold count.

Furthermore, the cecum was found to contain a TBC, TYMC, *coliform*, *E*.* coli*, *S*. *spp*., *E*.* spp*., and LAB. Figure 1 shows that the ZnNPs + P group significantly reduced all microbial populations in the broiler’s cecum, followed by the P and ZnNPs groups. However, the quantity of LAB exceeded that of the control group.* Salmonella *was absent from the cecum of the treatment group.

## Discussion

The antimicrobial and oxidative characteristics of ZnNPs arise due to their diminutive dimensions and their abundance of phenolic chemicals on the nanoparticle surface [40]. The active compounds may account for the advantageous impacts of NPs additions on avian performance, carcass characteristics, blood parameters, and microbiological health. Meanwhile, the amalgamation of ZnNPs enhanced antibacterial and antioxidant activities. Narayanan et al. [41] found that ZnNPs (40 µg/ml) had inhibitory diameters of 19 mm versus *Staphylococcus aureus* and 14 mm against *E*.* coli*. This demonstrated the effectiveness of ZnNPs in eliminating the harmful bacteria under test. The widths of the inhibitory zones of ZnNPs (50 µg/ml) on these bacteria were 18 and 16 mm [42]. These outcomes align with our findings. Hassani et al. [43] found that ZnNPs had MIC and MBC values between 158 and 325 µg/ml against 15 different strains of *P*.* aeruginosa*. The MIC was 50 µg/ml for *E*.* coli*, *B*.* subtilis*, and *S*.* aureus* and 25 µg/ml for *Vibrio cholera* and *Clostridium botulinum*.

The ZnNPs and probiotics showed extensive antifungal efficacy in the present investigation. Arciniegas-Grijalba et al. [44] discovered that ZnNPs could kill *Erythricium salmonicolor*, the fungus that causes pink illness, in the laboratory by blocking the growth of fungal mycelia. The advantageous effects of probiotics on avian performance may result from alterations in the gut environment and the enhancement of beneficial microbial immunity. Competitive activity diminishes dangerous germs and stimulates the immune system [23]. Probiotics establish beneficial bacteria in the intestine, thereby eliminating opportunities for harmful bacteria to thrive or proliferate. Moreover, probiotics promote the secretion of digestive enzymes, including galactosidase and amylase, thereby improving animal efficiency [45].

**Table 7.** Effect of ZnNPs, probiotics, and their interaction on dietary microbiota (total bacteria, yeast and molds, * Escherichia coli*, and *coliform*) presented (Log CFU/ml) in broiler during feeding period of 0–3 weeks.

Traits	Feeding period (weeks)	Treatments				*p value^1^*		
		Control	ZnNPs	P	ZnNPs + P	T	L	Q
Samples/Microbial count								
TBC	0	5.8 ± 0.2^a^	5.5 ± 0.4^ab^	4.8 ± 0.6^c^	4.6 ± 0.3^c^	0.002	0.153	0.039
	1	6.0 ± 0.1^a^	5.7 ± 0.2^ab^	5.1 ± 0.4^c^	4.9 ± 0.5^c^	0.032	0.462	0.371
	2	6.3 ± 0.2^a^	6.1 ± 0.2^a^	5.5 ± 0.4^b^	5.3 ± 0.2^c^	0.017	0.193	0.361
	3	6.7 ± 0.5^a^	6.5 ± 0.1^a^	5.9 ± 0.5^b^	5.9 ± 0.5^b^	0.012	0.372	0.014
TYMC	0	3.8 ± 0.1^a^	3.5 ± 0.2^ab^	2.8 ± 0.7^bc^	2.5 ± 0.8^c^	0.021	0.013	0.253
	1	4.0 ± 0.9^a^	3.7 ± 0.2^ab^	3.1 ± 0.4^c^	2.9 ± 0.8^cd^	0.029	0.023	0.049
	2	4.3 ± 0.1^a^	4.0 ± 0.3^ab^	3.4 ± 0.4^c^	3.1 ± 0.5^cd^	0.001	0.036	0.251
	3	4.8 ± 0.2^a^	4.5 ± 0.5^b^	3.8 ± 0.4^c^	3.6 ± 0.8^cd^	0.004	0.425	0.314
*E. coli*	0	2.2 ± 0.2^a^	2.0 ± 0.1^ab^	1.5 ± 0.6^bc^	1.3 ± 0.4^c^	0.009	0.148	0.316
	1	2.5 ± 0.6^a^	2.3 ± 0.2^ab^	1.9 ± 0.2^bc^	1.7 ± 0.3^c^	0.036	0.237	0.019
	2	2.9 ± 0.2^a^	2.6 ± 0.2^ab^	2.1 ± 0.5^bc^	1.9 ± 0.6^c^	0.027	0.315	0.713
	3	3.1 ± 0.2^a^	2.8 ± 0.1^ab^	2.3 ± 0.7^bc^	2.0 ± 0.8^c^	0.014	0.025	0.062
Coliform	0	2.9 ± 0.1^a^	2.7 ± 0.2^ab^	2.2 ± 0.1^bc^	1.9 ± 0.4^c^	0.006	0.072	0.324
	1	3.3 ± 0.3^a^	3.7 ± 0.2^ab^	2.5 ± 0.8^bc^	2.1 ± 0.1^c^	0.000	0.103	0.273
	2	3.6 ± 0.2^a^	3.4 ± 0.3^a^	2.9 ± 0.4^b^	2.7 ± 0.7^b^	0.015	0.097	0.168
	3	4.0 ± 0.2^a^	3.8 ± 0.1^a^	3.2 ± 0.3^b^	3.0 ± 0.7^b^	0.005	0.074	0.243

*E*.* coli* = *Escherichia coli;* P = probiotics 2 cm^3^; TBC, total bacterial count; TYMC: total yeast and mold count; ZnNPs = zinc nanoparticles 3 cm^3^; ZnNPs + P = ZnNPs 3 cm^3^ + P 2 cm^3^.

^a–d^Different letters within one row are significantly different (*p *< 0.05).

The combinations of ZnNPs and ZnNPs + P exhibited the highest LWG across the groups. At the same time, the treatments supplemented with ZnNPs, P, and ZnNPs had superior BWG values throughout the entire experiment. The beneficial effect may result from ZnNPs enhancing intestinal absorption by improving mucosal efficiency [46]. The enhanced adsorption capability of ZnNPs increases Zn bioavailability [47]. Previous studies have shown that adding ZnNPs to feed can increase LBW and FCR, lower the number of microbes in the gut, and boost the immune system [5]. Our outcomes align with those of Mahmoud et al. [48], who indicated that ZnNPs (10 ppm) significantly improved BWG and FCR in broilers. According to Fathi et al. [49], birds that were fed diets that contained ZnNPs gained significantly more body weight and had a lower FCR than birds that were fed a control diet. Numerous studies have confirmed that zinc-supplemented diets enhanced growth rate and feed effectiveness in broilers [50,51].

The improvement in certain carcass features in birds fed a ZnNPs + P diet may be ascribed to the antimicrobial properties of ZnNPs, which diminish the burden of harmful microbes and boost gut health [51]. Previous research demonstrated that dietary supplementation of ZnNPs (40–90 ppm) enhanced dressing percentage and carcass yield [52]. Additionally, Abd El-Hack et al. [5] suggested that the dressing yield of broiler chicken was substantially improved by dietary utilization of ZnNPs, specifically at a dose of 0.4 mg ZnNPs per kg of diet. Ashour et al. [53] discovered that a diet with ZnO-Nano-ALPE substantially enhanced broiler chicken dressing output, carcass quality, and giblet weight. The addition of ZnNPs-MLPE did not influence carcass features [8]. Furthermore, Mahmoud et al. [48] established that birds fed diets containing ZnNPs had significantly greater spleen and bursa weights compared to other groups. Abd El-Moneim et al. [54] observed no statistically significant alterations in the carcass attributes of broilers administered by bifidobacteria.

Our findings found that the nutritional treatment of ZnNPs + P, ZnNPs, and P significantly increased TP, albumin, globulin, calcium, immunoglobulins, enzymes, amylase, protease, and lipase levels compared to the control group while reducing TG, TC, LDL, VLDL, and MDA levels. Our outcomes were consistent with those of Abd El-Hack et al. [5], who demonstrated that ZnNPs markedly decreased chicken TC, LDL, and abdominal fat levels while elevating HDL values. Additionally, Mahmoud et al. [48] reported that the incorporation of 20 ppm of ZnNPs into the diet markedly reduced serum TG levels. The present results aligned with those of El-Bahr et al. [55], who showed that supplementation with ZnNPs at doses of 20 or 40 mg/kg diet decreased TC and TG while increasing HDL. In addition, Bashar et al. [56] noted that incorporating dietary minerals NPs lowered the amounts of AST, ALT, and TG in the blood. Moreover, the decrease in TC may result from zinc’s capacity to inhibit cholesterol absorption in the intestines and to promote the proliferation and viability of LAB, which decreases TC levels [57].

The elevation of immunity concentrations in blood in ZnNPs + P could be ascribed to the beneficial impacts of the tripartite mixture of ZnNPs and probiotics, which enhanced the immunity of birds by the additive nature of their antioxidant qualities. Conversely, the incorporation of ZnNPs into a smoker’’s diet resulted in elevated blood lipid levels, as reported by Fathi et al. [49]. Zinc plays a part in lipid enzymes, so a diet high in ZnNPs (20 mg/kg) raises blood cholesterol levels and increases SOD activity to get rid of free radicals [58]. Probiotics did not influence serum markers, with the exception of serum Ca and glucose [59]. Biogenic ZnO-NP synthesis initiates with the complexation of zinc ions facilitated by microbes and water molecules within an acidic environment. This involves the formation of a zinc aqua-hydroxo complex, which subsequently transforms into ZnO-NPs. This transformation is driven by the acceptance of electrons from deprotonated carboxyl groups of bioactive molecules (e.g., enzymes, peptidoglycan) released by the bacteria [12]. In certain instances, specific organic functional groups on the bacterial cell wall directly contribute to non-enzymatic ZnO-NP synthesis by reducing zinc ions. Heat-killed microbial cells release these organic molecules (acting as reducing agents) due to cell membrane rupture and lysis, further enhancing zinc ion reduction [8,31].

Our findings illustrated that the addition of ZnNPs and P to broiler diets significantly enhanced meat moisture, protein, and pH; reduced lipid content; and enhanced yellowness, juiciness, tenderness, and flavor compared to the control group. This outcome aligns with previous research, which suggests that the pH of the avian meat in this investigation, which ranged from 5.5 to 6.3, falls within the normal of 5.3 to 6.5 [60]. ZnNPs administered at a dosage of 0.2 mg/kg had a pH significantly higher than that of the control group. This aligned with Soeparno’s [61] findings, which indicated that supplementary zinc significantly elevated broiler muscle pH values. In contrast, Selim et al. [62] observed a 6.8% reduction in the pH of the thigh and breast muscles of broilers administered ZONPs. In comparison to the control, ZnNPs at 0.2 mg/kg exhibited no significant impact on color or overall acceptance. According to Selim et al. [62], ZONPs at concentrations of 40 or 80 ppm did not influence the color, texture, aroma, or overall acceptance of chicken meat.

The study revealed lower bacterial counts in the treated groups compared to the control group. ZnNPs + P showed superior performance in reducing TBC, TYMC, *E*.* coli*, and *coliform* counts. Additionally, the ZnNPs + P group significantly decreased the microbial population in the broiler’s cecum. LAB values exceeded those of the control group, and *Salmonella* was not detected in the treatment. Abd El-Moneim et al. [54] demonstrated the positive effects of probiotics on avian viability and gastrointestinal health. ZnNPs can influence avian metabolic efficiency and health due to their antibacterial and immune-modulatory properties [63]. Furthermore, Ahmadi et al. [64] suggested that higher concentrations of ZnNPs, specifically 30–80 ppm, could enhance broiler efficiency. ZnNPs can regulate nucleic acid and protein metabolism in broilers by amplifying the effects of growth hormone genes [65].

Studies have demonstrated that zinc oxide nanoparticles are effective against both “Gram-positive and Gram-negative bacteria” [42]. In comparison to the TBC, ZONPs at reduced doses (10 ppm) exhibited the most potent antibacterial efficacy. Certain investigators [66] assert that increased permeability, which significantly affects transport across the cell membrane, is the cause of bacterial cell death. The antibacterial properties of nanoparticles are primarily determined by their surface area and concentration, with the surface area and potent bactericidal impact increasing with reducing particle size [67]. In addition, Liu et al. [68] demonstrated that ZONPs might efficiently prevent the proliferation of pathogens such as *E*.* coli* and *Salmonella enteritidis*. Song et al. [69] found that antibiotic growth promotes feed-reduced *Lactobacillus*, potentially increasing the number of *Lactobacillus* strains in broilers. However, they also reduced the abundance of *Lactobacillus *sp. in the ileal digesta [17].

Aldal’in et al. [70] discovered that prodigiosin and ZnNPs help rabbits manage DNA damage, oxidative stress, and inflammatory responses, potentially alleviating the effects of heat stress. Furthermore, El-Shobokshy et al. [71] found that replacing inorganic zinc with zinc nanoparticles improved the body weight, body weight gain, and feed conversion ratio of male rabbits. Additionally, rabbits given ZnNPs showed improvements in histopathological findings, reproductive capacity, and oxidative parameters.

In summary, the study indicates that the antimicrobial activity of ZnNPs or *Bacillus* spp. mixture provided more beneficial bacteria in the intestinal cavity. In this sense, reducing pathogenic bacteria in the intestine may reflect a reduction in microbial load in meat quality after slaughtering. This feeding strategy may establish a stronger link between dietary practices and extended meat shelf life, contributing to a more sustainable and environmentally sound approach within the poultry sector.

## Conclusion

The combination of ZnNPs and probiotics showed synergistic effects, making it economically feasible. This synergy improved growth performance, blood parameters, meat quality, and antioxidant levels. The combination also exhibited superior antibacterial properties compared to using them individually. It reduced pathogenic microorganisms and increased LAB levels. This study suggests that incorporating ZnNPs and probiotics in broiler feed is a cost-effective and safe alternative to using high doses of zinc and antibiotics.

## List of abbreviations

BWG, body weight gain; FCR, feed conversion rate; FI, feed intake; GLM, the General Linear Model; HDL, high-density lipoprotein; LBW, low birth weight; MH, Mueller Hinton; MHA, Mueller–Hinton agar; MHB, Mueller Hinton Broth; MRS, de Man, Rogosa and Sharpe (MRS) agar; NPs, nano particles; NRC, National Research Council; PI, Proportional-Integral; SDA, Sabouraud dextrose agar; SD, Sabouraud Dextrose; SDB, Sabouraud Dextrose Broth; TBA, thiobarbituric acid test; TBC, total bacterial count; TVBN, the total volatile base nitrogen; TYMC, total yeast and mold count; ZnNPs, zinc nanoparticles; ZONPs, Zinc Oxide nanoparticles.
